# “The patient as teacher” - thematic analysis of undergraduate medical students’ experiences with an experiential learning project in palliative care

**DOI:** 10.1186/s12904-024-01570-9

**Published:** 2024-10-09

**Authors:** Stephanie Stocklassa, Susan Block, Piret Paal, Frank Elsner

**Affiliations:** 1 Department of Palliative Medicine, Medical Faculty, RWTH Aachen University, Pauwelstraße 30, Aachen, 52074 Germany; 2 Department of Psychosocial Oncology and Palliative Care, Harvard Medical School, Boston, USA; 3 Department of Ethnology, Institute of Cultural Studies, Tartu, Estonia, and Institute of Palliative Care, University of Tartu, Paracelsus Medical University, Salzburg, Austria

**Keywords:** Palliative care, Undergraduate medical students, Teaching, Experiential learning, Reflective writing

## Abstract

**Background:**

Experiential learning holds high potential for medical students’ education in palliative care. At RWTH Aachen University in Germany, medical students can participate in the course “The Patient as Teacher” offering a one-to-one exchange with a terminally ill patient over a period of several weeks complemented with four supervision sessions and writing of a reflective essay. The course had run from 2005 to 2020 before it was paused due to the Covid-19 pandemic. This study aimed to assess the course’s value as a palliative care teaching tool by investigating students’ motivation and experiences over the years 2005–2020.

**Methods:**

A stratified sample of 24 essays was taken from all submitted essays (*n* = 78), eight essays from the years 2005–2009, 2010–2014, and 2015–2020. Subsequently, a thematic analysis of the selected essays was conducted.

**Results:**

The students felt motivated by the opportunity to gain more experience in palliative care, to improve their communication skills and to decrease insecurities in interaction with terminally ill patients. They learned about the patient’s biography and medical history, and encountered physical, psychological, social, and spiritual dimensions of living with a life-limiting disease. Moreover, they experienced relationship building and communication with a terminally ill patient outside their role as future doctors. Ultimately, they considered their participation as a beneficial experience on both a personal and professional level.

**Conclusions:**

The course “The Patient as Teacher” presents a valuable tool for experiential learning in palliative care, which has elicited an unceasingly positive response among the students who participated over the years. It has facilitated medical students in overcoming insecurities in dealing with terminally ill patients and supported them in further developing their professional identity.

## Background

Given global demographic change and the rising rate of non-communicable diseases, the demand for comprehensive palliative care will continue to rise [1]. Therefore, the European Association for Palliative Care has argued that all medical professionals should be capable of providing appropriate palliative care as they will encounter patients in need of palliative care from primary care to tertiary hospitals [2]. Consequently, increased demands are placed on the education and training of medical professionals today. In this context, undergraduate medical students’ access to palliative care education has been significantly expanded at German universities over the past few decades [3, 4]. Since 2009, it has been required by law that all medical students in Germany are trained in the provision of palliative care (ÄApprO as of 03.07.2002, last amended in 31.07.2009, § 27 and supplement 15 to § 29 Sect. 3 sentence 2) [5]. Simultaneously, it has been acknowledged that practical training and clinical exposure are essential for teaching students how to interact adequately with terminally ill patients and their families [6]. Particularly, palliative care education and training needs to support students in developing appropriate attitudes towards caring for terminally ill patients [3]. Consequently, German universities have chosen various approaches to provide students with practical experience in the field of palliative care.

Since 2005, medical students at RWTH Aachen University have had the opportunity of participating in the course “The Patient as Teacher”, offering a one-to-one exchange over several weeks with a patient receiving palliative care. Following the model of experiential learning, the course aims to help students understand experiences of terminally ill patients through personal exchange. Taking part is voluntary for both patients and students, and students from all years may apply to attend the course. While the individual patient largely determines the course of the exchange, the student has no specific task or assignment. Therefore, students may temporarily distance themselves from their role as future health professionals and meet the patients on a purely personal level. This is to enable the students to gain a more holistic view on the consequences of terminal illness on the individual. While other programmes let students accompany doctors or volunteers at e.g. hospice or home care visits [7, 8], the uniqueness of the course “The Patient as Teacher” is offering students to meet terminally ill patients on their own. This creates a more authentic environment that allows students to practise their communication skills more effectively. The course is based on a teaching concept from Harvard Medical School, Center of Palliative Care [9]. To our knowledge, no comparable palliative care course has been described in the literature to date.

In particular, the course consists of three components: accompaniment of a terminally ill patient, supervision in small groups under psychological guidance, and writing of a reflective essay. The accompaniment of the patient comprises a total of 20 h over a period of four to six weeks, with the actual frequency and duration of the meetings being determined individually between the patient and the student. Typically, the course extends over 10 to 25 encounters. While the first meeting takes place on the hospital ward, subsequent meetings might also take place at a hospice or at the patient’s home after the patient’s discharge.

Supervision usually takes place at four meetings spread over the period of the course. Here, students receive input for personal reflection and help with potential problems in interaction with their patients. At the same time, the meetings offer an exchange of experiences with other students taking part in the course. At the end of the course, students are requested to hand in a twenty-pages reflective essay on their experiences. Students have the full freedom regarding the form and content of their essay, which it is seen as an invitation for reflection and evaluation. Ultimately, students do not receive a formal grade for their participation.

The course “The Patient as Teacher” had run for a period of sixteen years before it was paused at the beginning of the Covid-19 pandemic. Over this period, the students’ interest in participating constantly exceeded the availability of placements, which led to long waiting lists. In 2009, the course “The Patient as Teacher” won the Palliative Medicine Promotion Award of the German Society for Palliative Medicine.

There is a broad consensus that medical students benefit from experiential learning and reflective writing in the palliative care learning process. Particularly, one-to-one encounters with patients reveal the social, psychological and spiritual dimensions of illness to the students [10–13]. Simultaneously, authors underline that reflective training supports medical students in forming their professional identity [10, 11, 14]. In particular, comprehensive palliative care provision requires competencies of respectful and empathetic communication as well as professional behaviour with regards to one’s personal and professional values [2, 15]. Likewise, experiential learning offers the chance to positively influence students’ attitudes towards palliative care by paying attention to the hidden curriculum (implicit changes in students’ attitudes, beliefs, and values due to extracurricular experiences and observations) [16, 17]. While it is understood that first encounters with terminally ill patients can be distressful, it is important to provide a safe learning environment in which students can experience positive relationships, guidance and self-efficacy [13, 14].

Analysing students’ experiences within the course “The Patient as Teacher” may provide valuable implications for experiential learning in palliative care. Simultaneously, it is unknown whether the expansion of palliative care education had an impact on students’ motivation and experience with the course over the years. Therefore, this study aims to investigate students’ motivation and experiences within the course “The Patient as Teacher” by analysing students’ reflective essays from 2005 to 2020. In this respect, particular attention shall be paid to potential changes in students’ perceptions over the years.

## Methods

In the period 2005–2020, a total of 114 medical students participated in the course (95 female and 19 male students). Of these, a total of 78/114 (68%) students submitted a reflective essay (65 female and 13 male students). For this study, a sample of 24/78 (31%) essays was taken from all essays using stratified randomisation weighted by the number of essays submitted per year, eight essays each from the years 2005–2009, 2010–2014 and 2015–2020, subsequently referred to as group A, group B, and group C. Ultimately, the essays of 21 female and 3 male students were included for analysis. The average length of essays was 7361.50 words (SD = 1655.25).

An assessment by the Medical Ethics Committee of RWTH Aachen was obtained in 2022 for this study stating that there are no ethical or occupational law concerns regarding the research project (EK 258/22). Before participation, all students gave their informed consent for their anonymised essays to be used for future studies. At the same time, it was emphasized that the students could withdraw their consent at any time without any negative consequences or the need for justification. All essays were kept anonymous and confidential, and participants were assigned a random identification number (e.g. C5) to warrant protection of identity. Those numbers were included with the quotes from the individual essay to illustrate the breadth of data.

The analysis was carried out using MAXQDA (version 2022.2.0) by a female medical student with a background in health science and nursing (corresponding author), who had not been involved in the course otherwise, with guidance from a tenured professor at RWTH Aachen university (co-author). Thematic analysis was chosen to gain an understanding of the students’ motivations, perceptions, and experiences as expressed in their reflective essays. The data analysis was guided by Braun and Clarke’s (2022) “Reflexive Thematic Analysis approach” [18, 19]. After familiarization with the data (step 1), initial codes were generated, and all relevant data collated to each code (step 2). Then, corresponding codes were gathered into potential themes (step 3). Next, the themes were reviewed whether they matched the initial codes as well as the dataset. The themes were refined and specified creating the final thematic ‘map’ of the analysis (step 4). Subsequently, the naming of themes was reviewed to unfold the overall story of analysis (step 5). Ultimately, illustrative quotes were selected, and the report was written while relating the analysis back to the research question and literature (step 6). The analysis was carried out in German language and only the final themes and corresponding text passages were translated into English under the guidance of a native speaker.

## Results

The analysis revealed four main themes concerning students’ experiences within the course, namely *motivation*, the *patient’s story*, the *interaction* with the patient, and *self-reflection*. In particular, *motivation* concerned students’ expectations of the course and reasons for participating. The *patient’s story* referred to the personal and medical history of the patient as well as his or her individual constraints, needs, and wishes. *Interaction* mainly concerned aspects of communication and relationship-building. Lastly, *self-reflection* involved revisiting personal feelings and boundaries, felt notions of responsibility, and consequences of experiences within the course for the students’ professional self-image as prospective doctors (Table 1). Finally, some students gave feedback on the course in their essays, which will be summarised at the end.

**Table 1:** Main themes and associated key codes as identified by analysis of students’ essays

Main theme	Key codes
Motivation	Spending more time with a patient
	Exploring new learning concept
	Encountering patients outside their role as medical students
	Filling gaps of regular curriculum
	Reducing insecurities in dealing with terminally ill patients
	Improving communication skills
	Taking on new challenge
Patient’s story	Personal biography
	Medical history
	Dimensions of living with a life-limiting illness (physical, psychological, social and spiritual)
	Dreams and wishes
	Concept of quality of life
Interaction	Communication (conversation style, sharing feelings etc.)
	Relationship-building
	Closeness and distance
	Defining their own role
	Overcoming obstacles
	Saying goodbye
Self-reflection	Upcoming feelings (happiness, sadness, insecurity etc.)
	Personal boundaries
	Own life and personal values
	Professional self-image as future doctors

### Motivation

Concerning their motivation to participate in the course, students most frequently reported taking the opportunity to spend more time with a patient than possible in their future working life. Specifically, students felt that the course offered them the opportunity to experience a more in-depth exchange with a patient, as one student reported:

I know that I cannot and will not get involved with every one of my future patients in the way I was able to do with [patient’s name] in this course. Yes, it was a privilege for me to be able to accompany a person in such a special way and so intensively in terms of time (C3, female).

Simultaneously, students appreciated the concept of experiential learning and, in particular, being in contact with patients without a specific teaching assignment and outside of their role as doctors. In this context, one student noted:

I have certainly heard the medical facts in a lecture before and I can research them relatively easily; however, the personal account and experience of the disease with all its aspects (i.e. also psychological, mental and spiritual) is something that cannot be taught so easily in conventional academic courses (C8, female).

For many students, the course represented their first one-to-one encounter with a terminally ill person. Many students lamented that insufficient attention was paid to palliative care in their regular course work. Likewise, they felt that the regular curriculum did not sufficiently prepare them for working with terminally ill patients. The students hoped that their participation in the course would help to improve their communication skills and reduce their insecurities when dealing with seriously ill patients and death. Generally, the students perceived their participation as a challenge as they were not familiar with the teaching concept and, simultaneously, did not have a clear idea of what they would be encountering. Accordingly, many of the students expressed concerns and fears about what to expect from the course and whether they were up to the challenge:

As soon as I had the acceptance, my thoughts began to circle: Can I do it at all? What did I sign up for here? (B6, male).

Ultimately, many students took part out of curiosity or on the recommendation of other students.

### Patient story

The course enabled students to gain insight into their patients’ personal biography as well as their medical history. Due to the personal character of the meetings, the students got to know the patient’s social environment and often encountered the patient’s relatives as well. In this context, many students reflected on the social dimension of disease and the importance of social support for the patient. Moreover, students could discover their patients’ distinct needs and develop an understanding for their patients’ experiences of living with a life-limiting illness:

I was allowed to get to know a person in a very unbiased way, to get to know a part of his inner being without much ado, to share happy and bitter moments, to meet family and friends, to experience conversations, memories, cohesion, quarrels, hope and suffering. (C7, female).

Particularly, students reported that they got to know their patients’ dreams and wishes and learned what quality of life could mean for a terminally ill person:

It always warmed my heart to hear her talk about strawberry ice cream cups with shining eyes. In those moments I really forgot that she was sick - that is how much she could enjoy the little things (A7, female).

Some students have also learned about the spiritual needs of the patient. At the same time, students witnessed the patients’ aggravating physical limitations as well as the emotional burden linked to the progression of disease:

Illness puts the sufferer in a state of physical and mental inconstancy: the sufferer has to constantly redefine himself and explore which abilities and things can still be carried out in everyday life. Phases of hope alternate with phases in which hope seems to be exhausted (B2, male).

While some students paid a last visit to their patient after his or her death, none of the students were present when their patient died.

### Interaction

According to the students, interacting with their patients required skills in communication and relationship-building. In order to gain their patients’ trust, finding the most appropriate conversation style was crucial. Jointly, students could practise exchanging views, sharing personal feelings, expressing empathy, and setting boundaries. Some students aimed to make regular appointments and create shared rituals (e.g. going for a walk or sharing a cup of coffee in the patient’s kitchen) to build up a relationship towards their patient. Generally, students aimed to find common interests with their patients. Many students reported they were surprised at how quickly they formed a trustful bond with their patient:

You meet a […] person in a closed room, with limited time, and despite this, or precisely because of this, a climate of trust builds up relatively quickly […] I have the feeling that you don’t achieve this closeness so easily and quickly with people you will see again or with whom you have connections (B6, male).

At the same time, some students found it difficult to connect with their patient:

It took a lot of strength and commitment to establish and maintain contact with her. During the whole time, I tried to recognise her fears and needs and to help her cope. Initially, this seemed almost impossible due to the patient’s resistance. In the course of the conversations, however, an increasingly trusting relationship developed between [patient’s name] and me. (A4, female).

In this context, students reflected on the appropriate degree of closeness and distance towards their patient:

It helped me a lot to be aware of my motivation and the goal of a meeting beforehand. Because these two points only became fully apparent to me in the course of the course, due to [the course’s] concept and my personal characteristics, I saw a challenge in sensing and finding this boundary. (B5, female).

Whereas many students initially feared to burden the patient with their presence, they later described the mutual benefit of the encounter:

In the beginning I felt out of place. I thought I was disturbing the family, who surely had so many things to discuss. This feeling disappeared very quickly after I realised how much my patient and his wife looked forward to my visits. (A3, female).

Some students thought that their patients valued sharing their thoughts with an unbiased person. Others reported they could provide emotional support when their patient was in distress:

I knew that I could not relieve him of the burden of dying, but that perhaps through my visits I could carry a little more diversion and entertainment into his environment and lessen the loneliness and isolation to which he was subjected in that room. (A2, female).

At the same time, students repeatedly mentioned being unsure of their own role in interaction with the patient:

What am I doing here anyway? Where is this supposed to lead? […] What is the justification for my presence?” (A8, female). “What is my goal? My purpose in our conversations? The purpose of my visits to her? (C1, female).

In this context, students frequently reported that their role and purpose was implicitly defined by the patient and his or her needs:

Overall, the course gave me the opportunity to search for and question my role through my patients […] Thus, from my perspective throughout the course, I was in varying proportions a learner, an independent, a confidant and a listener, who was also asked for assessment or advice at times (B5).

In addition, the students reported on some obstacles in interacting with their patients. Occasionally, they experienced difficulties in scheduling meetings with their patient. Simultaneously, several patients were limited in their ability to communicate due to their illness. In some cases, only non-verbal communication was possible at times. Moreover, some students did not know how to react to the patient’s statements:

And again the sentence: “My days are numbered”. These words sound like something out of a drama, so unrealistic and useless for daily use. Is that why it makes me feel uneasy? There is an end in sight, negative associations. Why did she tell me that? Was it an accepting ‘ok’ or a desperate ‘why’? What did she expect me to answer? (B8, female).

Similarly, some students faced issues of miscommunication and conflict they had to solve. For instance, when the expectations of patients and students diverged. In this context, many students indicated that they found the input from the supervision sessions very helpful. Ultimately, the concerned students found ways to solve the tension between the different parties:

I have learned how important it is to address problems directly with those involved. In this way, misunderstandings can be quickly resolved and people can deal with each other in a completely different way (B4, female).

For the majority of students, saying goodbye to their patient formed the most challenging part of the course:

I would have liked to be with her until the end. Perhaps it was right to distance myself beforehand and to consciously end the accompaniment. Sometimes it feels wrong to have the opportunity to choose when to say goodbye, while others have no choice. (C7, female).

This farewell could likely be the final one. This woman came very close to me, or rather I let her come very close to me, and it is not easy for me to say goodbye today. It is a gentle regret mixed with the knowledge that this is the course of life. (B1, female).

In some cases, the patient died before the actual end of the course. Depending on how established the relationship between student and patient was at that point, this experience could be very distressing for the concerned student:

How could this happen so quickly? I felt terrible because I had always put off saying goodbye […]. A conscious farewell to [patient’s name] at the end of the internship would have been enriching for both of us. (A7, female).

I was particularly affected by the death at that time, more than I would have expected. I had met [patient name] as a palliative care patient, so I knew that he could die at any time. But somehow I just didn’t expect it at the time. (C2, female).

### Self-reflection

Students’ participation raised many feelings among them, which they reflected on in their essays:

Not only during the course, but also while I was writing this text, I experienced a rollercoaster of emotions. I was happy, joyful, sad, despairing, angry and stunned and much more than could be put into words easily (B7, female).

At the beginning of the course, many students felt insecure as they had no clear picture of what was expected from them or unsure how to approach a terminally ill patient. Occasionally, students were uncomfortable with the thought of taking up the patient’s time:

I suddenly felt a very bad conscience to be there at all and to let such a sick person gift me with time. (B5, female).

However, those feelings generally diminished over the period of the course once they got to know their patients better and built up a relationship towards them. Being met with openness and kindness, many students developed a sense of responsibility towards their patients. The more the students built up closeness and trust with their patient, however, the more worried they became about the thought of parting and the possible death of their patient:

At some point I was afraid that his wife would pick up the phone and tell me that her husband had died. I didn’t want to stumble into such a situation. (A6, female).

At the same time, some students felt rejected and disappointed as they faced difficulties in connecting with their patient, as mentioned above. Generally, interacting with their patient required many students to become aware of their personal boundaries. Students reported that they allowed themselves to be more personally involved than they would be in their role as doctors:

Our relationship takes on almost family-like structures. (B1, female).

Simultaneously, those students stated they had been able to take away more from the encounter by putting off the ‘white coat’. As many students felt sad for leaving their patient at the end of the course, some were too afraid to say goodbye:

Distancing and saying goodbye is one of the essential components of this course. I didn’t succeed, but you learn more from mistakes in the end. (B8, female).

Those students who were informed about the death of their patient needed some time to come to terms with what had happened:

Sometimes my thoughts still wander to the people whose difficult time and sad loss I witnessed, and I wonder how they are doing now. However, these questions arise less and less in me as time goes by (A2, female).

For many students, the exchange with their patient formed an occasion to reflect on their own lives and personal values:

Where would I be in 60 years? Will I be able to look back on my life happily? What losses will shape my life? (C5, female).

It is banal, but it is known that there is a fine difference between knowing things and comprehending things. I knew that death makes the time we have in our lives valuable. It was only in the course of my encounter with [patient name]. that I came to comprehend it. (C6, female).

Over the period of the course, many of the students rethought their professional self-image as future doctors. On the one hand, students witnessed how their patients felt concerning their caretakers’ behaviour towards them, including potential shortcomings. As future doctors, the students formed the wish to take time for their patients and to listen closely to them, despite all adversities of clinical routine:

By now, I find it more and more unbelievable how the simplest rules of interpersonal interaction can so often be lost in the clinical routine. […] I have noticed how difficult and important it is to communicate properly with patients in order to build up a relationship of trust (A7, female).

I believe that a doctor who is very brilliant professionally but not very empathetic or caring as a human being can achieve little cooperation with a patient (B3, female).

On the other hand, students drew conclusions based on the needs and emotional experience of their patient that they may have assessed differently beforehand:

When I listened to [patient name], I was surprised that he himself said that he did not know exactly what cancer meant. He also expressed his frustration that he did not understand the doctors’ letters […]. He felt overlooked, even though it was about HIS disease (C3, female).

Moreover, the students learned to respect the patient’s autonomy:

It is not always easy to understand the patient’s will, and it is difficult for a doctor to respect the patient’s autonomy without […] getting into a conflict of conscience of his own. […] So part of the doctor’s task is to find a balance between the patient’s will and what is right from a medical point of view. (A1, male).

Generally, the students recognised the value of palliative care:

For me, the medical profession took on a new dimension during the course. […] I learned the difference between ‘nothing more can be done’ and ‘it can no longer be cured’ (B7, female).

### The students’ verdict

Overall, the students perceived their participation as a valuable experience which was enriching on both a personal and professional level. Moreover, students commented on the value of supervision when facing difficulties in interaction with their patient. Likewise, students perceived the reflective essay as a valuable tool to process their experiences. At the same time, a few students also expressed criticism of the course. First, some students lamented a lack of structure and plannability as their participation caused a time conflict with their classes and exams. Second, some students struggled with the open character of the course and would have preferred to receive a more predefined assignment. Third, a few students mentioned difficulties in writing the reflective essay as they were not familiar with the concept:

Writing the report proved to be a great challenge for me. It took me quite a few attempts to write the report. This made me realise that I seem to have problems with such a task. I have never written so many pages on one topic. (B8, female).

Overall, the students drew a positive conclusion from their participation:

I am convinced that the experience I gained will be invaluable for me later on, regardless of the field I will work in one day. (A1, male).

For me, this course was a great preparation. Preparation for what? For life, I would say. For becoming a doctor and staying human (A8, female).

## Discussion

For more than 15 years, medical students have maintained a high level of motivation for the course “The patient as teacher” despite their intense curriculum. Motivated by a lack of expertise in dealing with terminally ill patients, students considered their participation a valuable and inspiring experience both on a professional and personal level. Although the students were not familiar with the course format, they approached the patient encounter with an open mind and adapted quickly. Offering a self-led learning experience, medical students could reduce insecurities and train their interpersonal skills which is expected to benefit their overall performance in palliative care [20, 21]. Overall, the course offered students a more holistic picture of the existential impact of terminal illness on the individual including social, psychological, and spiritual factors of disease.

Students’ experiences illustrate how experiential learning in palliative care can help building up students’ confidence in communicating with terminally ill patients. By offering students to spend time with palliative care patients by themselves they form a trusting relationship which helps students understand the patients’ experiences and needs. At the same time, many students had great difficulties saying goodbye after weeks of contact. In this context, supervision sessions represented a valuable tool to help students cope with their situation. Another lesson from students’ experiences is how experiential learning may positively influence students’ vision on end-of-life care. Moreover, the course made students rethink their self-image as doctors including their general behaviour towards patients as well as dealing with death and dying.

Nevertheless, some students also expressed criticism on “The patient as teacher”. Concerning the open character of the course, some students lamented the limited capacity for planning ahead. Likewise, some students missed having a defined assignment, which made them feel uncertain about their own role within the course. In this context, too, the students found the supervision sessions very helpful. In addition, the results of our study suggest that German students find reflective writing challenging. Although widely applied in other countries such as the United Kingdom, reflective writing is not standard at German universities [22]. While many students in this study appeared to be naturally self-reflective, others mentioned difficulties in the writing process, which could also be seen in the rather descriptive character of those essays. Similarly, some students might not have handed in their essay because they did not feel up to the task. It might therefore be useful to provide medical students with guidelines for reflective writing to facilitate the process and deepen students’ learning experience. Simultaneously, reflective skills will enhance students’ capacity for professional development [12].

The analysis revealed that students’ motivations to participate have remained unchanged over the years. On one hand, the course content’s relevance has not diminished. On the other hand, students’ insecurities in caring for palliative patients have remained despite the continuous expansion of the core curriculum. Studies in other European countries have yielded comparable findings [21, 23]. One reason might be that young adults are generally less likely to have experienced losses at this stage of their life. While the variety of palliative care teaching formats has increased over the last years (bedside-teaching, e-learning, simulated patients etc.), many German universities do not offer elective courses on palliative care at all [22]. Our findings underline the importance of experiential training as a tool to build up confidence for palliative care provision among medical students, which they should be offered to train besides their timely-limited curriculum [24].

It is striking that 83% of participants were female. While the university’s internal statistics show that the majority of medical students over the last ten years have been female, this does not adequately explain the large discrepancy in the gender distribution of participants. However, research suggests that female students may hold a more positive attitude towards palliative care than male students [25, 26] which could make female students more likely to take additional classes in palliative care.

As this study underlines the importance of supervision sessions in experiential learning courses, future research shall focus on how to support students in contextualizing their experiences. Moreover, studies are needed on how to effectively implement reflective writing as a learning tool for German medical students.

### Limitations

There are certain limitations to this study that need to be considered. The analysis was conducted by one person only which makes the results more susceptible to confirmation bias. Due to the descriptive nature of some of the reports, the overall depth of the analysis was variable. While the key themes arose in all analysed essays, the data needed to allow for a more in-depth analysis of the grounded theory approach (to identify underlying motives of observed patterns). Although participation was completely voluntary and no formal mark was given, it is not clear how truthful students were concerning their experiences and reflections as they might have intended to meet certain expectations. Reflective essays as written text are arguably helpful in restructuring consciousness [27]. However, the responsive orientation of written self-expression makes it debatable whether the writing for a course contains merely personal values and experiences or whether they represent socially desirable responses [28]. Yet, Ong (2002) suggests that any finished written text becomes removed from its author. This creates an independent discourse, and the text thus becomes a source for an independent analysis [27]. Lastly, 32% of the students who took part in the course did not hand in their reflective essay. While some students might have encountered time conflicts with their regular curriculum, others might have felt overchallenged with the task.

## Conclusion

“The Patient as Teacher” represents a highly valued palliative care learning tool among German medical students demonstrating the added value of experiential learning in palliative care education. It offers students a more holistic picture of the existential impact of terminal illness on the individual including social, psychological, and spiritual factors of disease while positively impacting medical students’ vision on end-of-life care. Moreover, it supports the students in overcoming insecurities in dealing with terminally ill patients and helps them to further develop their professional identity. Overall, “The Patient as Teacher” is a valuable and inspiring experience for medical students both on a professional and personal level.

## Data Availability

No data or material can be made available due to privacy and ethical concerns. Those interested may contact the corresponding author for further information.

## References

[1] World Health Organisation [Internet]. Palliative Care. Key facts. 2020. https://www.who.int/news-room/fact-sheets/detail/palliative-care. Accessed 13 May 2023.

[2] Gamondi C, Larkin L, Payne P. Core competencies in palliative care: an EAPC White Paper on palliative care education – part 2. Europ J Palliat Care. 2013;20(3).

[3] Ilse B, Alt-Epping B, Kiesewetter I, Elsner E, Hildebrandt J, Laske A, et al. Undergraduate education in palliative medicine in Germany: a longitudinal perspective on curricular and infrastructural development. BMC Med Educ. 2015. 10.1186/s12909-015-0439-6. 26383546 10.1186/s12909-015-0439-6 PMC4574529

[4] Elsner F, Ohlmeier L, Neukirchen M, Scherg A. Palliative care in medical education as QB13 and beyond-a teaching survey. [Palliativmedizin in Der Ärztlichen Ausbildung als QB13 und darüber hinaus–eine Lehrumfrage]. Z Palliativmed. 2018. 10.1055/s-0038-1669266.

[5] Elsner F. The new curriculum for students of the society for palliative medicine. [Das Neue DGP-Curriculum für Studierende]. Z Palliativmed. 2009; 182.

[6] Boland JW, Barclay S, Gibbins J. Twelve tips for developing palliative care teaching in an undergraduate curriculum for medical students. Med Teach. 2019. 10.1080/0142159X.2018.1533243. 30689479 10.1080/0142159X.2018.1533243

[7] Stecho W, Khalaf R, Prendergast P, Geerlinks A, Lingard L, Schulz V. Being a hospice volunteer influenced medical students’ comfort with dying and death: a pilot study. J Palliat Care. 2012. 10.1177/082585971202800304. 23098013

[8] Shunkwiler SM, Broderick A, Stansfield RB, Rosenbaum M. Pilot of a hospice-based Elective to learn comfort with dying patients in Undergraduate Medical Education. J Palliat Med. 2005. 10.1089/jpm.2005.8.344. 15890045 10.1089/jpm.2005.8.344

[9] Block SD, Billings JA. Learning from the dying. N Engl J Med. 2005;353(13):1313. 16192474 10.1056/NEJMp048171

[10] Braun UK, Gill AC, Teal CR, Morrison LJ. The utility of reflective writing after a palliative care experience: can we assess medical students’ professionalism? J Palliat Med. 2013. 10.1089/jpm.2012.0462. 23937062 10.1089/jpm.2012.0462 PMC3822362

[11] Fins J, Gentilesco B, Carve A, Lister P, Acres C, Payne R et al. Reflective practice and palliative care education: a clerkship responds to the informal and hidden curricula. Academic Medicine: J Assoc Am Med Coll. 2003; 10.1097/00001888-200303000-00015 10.1097/00001888-200303000-00015 12634214

[12] Morrison LJ, Thompson BM, Gill AC. A required third-year medical student palliative care curriculum impacts knowledge and attitudes. J Pal Med. 2012. 10.1089/jpm.2011.0482. 10.1089/jpm.2011.0482 22686121

[13] Roji R, Noguera-Tejedor A, Pikabea-Díaz F, Carrasco JM, Centeno C. Palliative care bedside teaching: a qualitative analysis of medical students’ reflective writings after clinical practices. J Palliat Med. 2017. 10.1089/jpm.2016.0192. 27754747 10.1089/jpm.2016.0192

[14] Boland JW, Dikomitis L, Gadoud A. Medical students writing on death, dying and palliative care: a qualitative analysis of reflective essays. BMJ Support Palliat Care. 2016. 10.1136/bmjspcare-2016-001110. 27486145 10.1136/bmjspcare-2016-001110

[15] Pieters J, Dolmans DHJM, Van den Beuken-van Everdingen MHJ, Warmenhoven FC, Westen JH, Verstegen DML. A national palliative care competency framework for undergraduate medical curricula. Int J Environ Res Publ Health. 2020. 10.3390/ijerph17072396. 10.3390/ijerph17072396 PMC7177526 32244658

[16] Billings ME, Engelberg R, Curtis JR, Block S, Sullivan AM. Determinants of medical students’ perceived preparation to perform end-of-life care, quality of end-of-life care education, and attitudes toward end-of-life care. J Palliat Med. 2010. 10.1089/jpm.2009.0293. 20178433 10.1089/jpm.2009.0293 PMC2883506

[17] Gaufberg EH, Batalden M, Sands R, Bell SK. The hidden curriculum: what can we learn from third-Year Medical Student narrative reflections? Acad Med. 2010. 10.1097/ACM.0b013e3181f57899. 20881818 10.1097/ACM.0b013e3181f57899

[18] Braun V, Clarke V. Using thematic analysis in psychology. Qual Res Psych. 2006. 10.1191/1478088706qp063oa.

[19] Braun V, Clarke V. Supporting best practice in reflexive thematic analysis reporting in palliative medicine: a review of published research and introduction to the reflexive thematic analysis reporting guidelines (RTARG). Palliat Med. 2024. 10.1177/02692163241234800. 38469804 10.1177/02692163241234800 PMC11157981

[20] Weber M, Braun J, Schildmann J. Effects of a ninety-minute teaching module for fourth-year medical students on a palliative care ward with student–patient encounter. J Palliat Med. 2011. 10.1089/jpm.2011.0025. 21767167 10.1089/jpm.2011.0025

[21] Wells G, Youssef E, Winter R, Wright J, Llewellyn C. Medical student confidence in care of the dying and their family: a systematic review. BMJ Support Palliat Care. 2020. 10.1136/bmjspcare-2019-001977. 31919103 10.1136/bmjspcare-2019-001977

[22] Ohlmeier L, Scherg A, Ilse B, Elsner F. Status of palliative care education in Germany: a survey of medical faculties in 2018. Schmerz. 2021. 10.1007/s00482-021-00536-7. 33576863 10.1007/s00482-021-00536-7

[23] Pieters J, Dolmans DHJM, Verstegen DML, Warmehoven FC, Courtens AM, Van den Beuken-van Everdingen MHJ. Palliative care education in the undergraduate medical curricula: students’ views on the importance of, their confidence in, and knowledge of palliative care. BMC Palliat Care. 2019. 10.1186/s12904-019-0458-x. 31455326 10.1186/s12904-019-0458-x PMC6712798

[24] Boland JW, Brown MEL, Duenas A, Finn GM, Gibbins J. How effective is undergraduate palliative care teaching for medical students? A systematic literature review. BMJ Open. 2020. 10.1136/bmjopen-2019-036458. 32912945 10.1136/bmjopen-2019-036458 PMC7482461

[25] Miltiades HW. University Students’ attitudes toward palliative care. Am J Hos Palliat Care. 2019. 10.1177/104990911987691. 10.1177/1049909119876911 31537081

[26] Dimoula M, Kotronoulas G, Katsaragakis S, Christou M, Sgourou S, Patiraki E. Undergraduate nursing students’ knowledge about palliative care and attitudes towards end-of-life care: a three-cohort, cross-sectional survey. Nurs Edu Tod. 2019. 10.1016/j.nedt.2018.11.025. 10.1016/j.nedt.2018.11.025 30554033

[27] Ong WJ. Orality and literacy. 2nd ed. London: Routledge; 2002.

[28] Paal P. Written cancer narratives [dissertation]. Helsinki (FI): University of Helsinki; 2011.

