# Correction: “The patient as teacher” - thematic analysis of undergraduate medical students’ experiences with an experiential learning project in palliative care

**DOI:** 10.1186/s12904-025-01693-7

**Published:** 2025-02-27

**Authors:** Stephanie Stocklassa, Susan Block, Piret Paal, Frank Elsner

**Affiliations:** 1 Department of Palliative Medicine, Medical Faculty, RWTH Aachen University, Pauwelstraße 30, Aachen, 52074 Germany; 2 Department of Psychosocial Oncology and Palliative Care, Harvard Medical School, Boston, USA; 3 Department of Ethnology, Institute of Cultural Studies, Tartu, Estonia, and Institute of Palliative Care, University of Tartu, Paracelsus Medical University, Salzburg, Austria

**Correction: BMC Palliat Care 23, 239 (2024)**

10.1186/s12904-024-01570-9

In this article [1] the ‘Authors’ contribution’ section was incorrectly given as' FE and SS were responsible for the study as well as the analysis and interpretation of findings. SB and PP were involved in drafting and revising the manuscript. All authors gave final approval to this manuscript.’ but should have been' FE and SS were responsible for the study as well as the analysis and interpretation of findings. **SS drafted the manuscript and SB and PP were involved in revising the manuscript.** All authors gave final approval to this manuscript’.

The original article has been updated.
